# Takayasu’s arteritis associated with eosinophilic gastroenteritis, possibly via the overactivation of Th17

**DOI:** 10.1186/s13099-018-0251-z

**Published:** 2018-06-12

**Authors:** Mikihiro Fujiya, Shin Kashima, Yuya Sugiyama, Takuya Iwama, Masami Ijiri, Kazuyuki Tanaka, Keitaro Takahashi, Katuyoshi Ando, Yoshiki Nomura, Nobuhiro Ueno, Takuma Goto, Kentaro Moriichi, Yusuke Mizukami, Toshikatsu Okumura, Junpei Sasajima, Daisuke Fujishiro, Kensaku Okamoto, Yuichi Makino

**Affiliations:** 1 Division of Gastroenterology and Hematology/Oncology, Department of Medicine, Asahikawa Medical University, 2-1 Midorigaoka-higashi, Asahikawa, Hokkaido 078-8510 Japan; 2 Division of Metabolism and Biosystemic Science, Department of Medicine, Asahikawa Medical University, Asahikawa, Japan

**Keywords:** Takayasu’s arteritis, Eosinophilic gastroenteritis, The overactivation of TH17

## Abstract

**Background:**

Takayasu’s arteritis (TA) is a large-vessel vasculitis pathologically characterized by granulomatous necrotizing vasculitis with giant cells. Although the cause of TA is still unclear, genetic factors as well as immunological abnormalities, particularly the overactivation of Th1 and Th-17, are considered to play important roles in the pathogenesis of this disease. Eosinophilic gastroenteritis (EGE) is a type of refractory inflammation in which numerous eosinophils infiltrate the inflammatory area. It is known that the overactivation of Th2 is associated with the pathogenesis of EGE, although the cause of EGE is still unclear. The immunological abnormalities in TA are therefore thought to be different from those in EGE. To date, no cases of complication of TA and EGE have been reported.

**Case presentations:**

An 18 year-old female was diagnosed with EGE and treated with prednisolone. At 6 months after completion of the treatment, the patient experienced chest pain, and was diagnosed with TA. TH1 and TH17 immunity are thought to be involved with TA, while TH2 are considered to be involved with EGE. In this case, the expression of IL-17 mRNA in the colon mucosa greatly decreased after prednisolone treatment for EGE.

**Conclusions:**

This is the first report of TA complicated with EGE, and the overactivation of TH17 is considered to be associated with the pathogenesis of these two diseases.

## Background

Takayasu’s arteritis (TA), which was first reported by Mikito Takayasu, is a large-vessel vasculitis pathologically characterized by granulomatous necrotizing vasculitis with giant cells [1]. Although the cause of TA is still unclear, genetic factors as well as immunological abnormalities, particularly the overactivation of Th1 and Th-17, are considered to play important roles in the pathogenesis of this disease [2, 3].

Eosinophilic gastroenteritis (EGE) is a type of refractory inflammation in which numerous eosinophils infiltrate the inflammatory area. It is known that the overactivation of Th2 is associated with the pathogenesis of EGE, although the cause of EGE is still unclear [4]. The immunological abnormalities in TA are therefore thought to be different from those in EGE. To date, no cases of complication of TA and EGE have been reported.

We describe the first case of TA that sequentially developed after EGE treatment in which overactivation of Th17 was thought to be associated with the pathogenesis of both TA and EGE.

## Case presentation

**Fig. 1:**
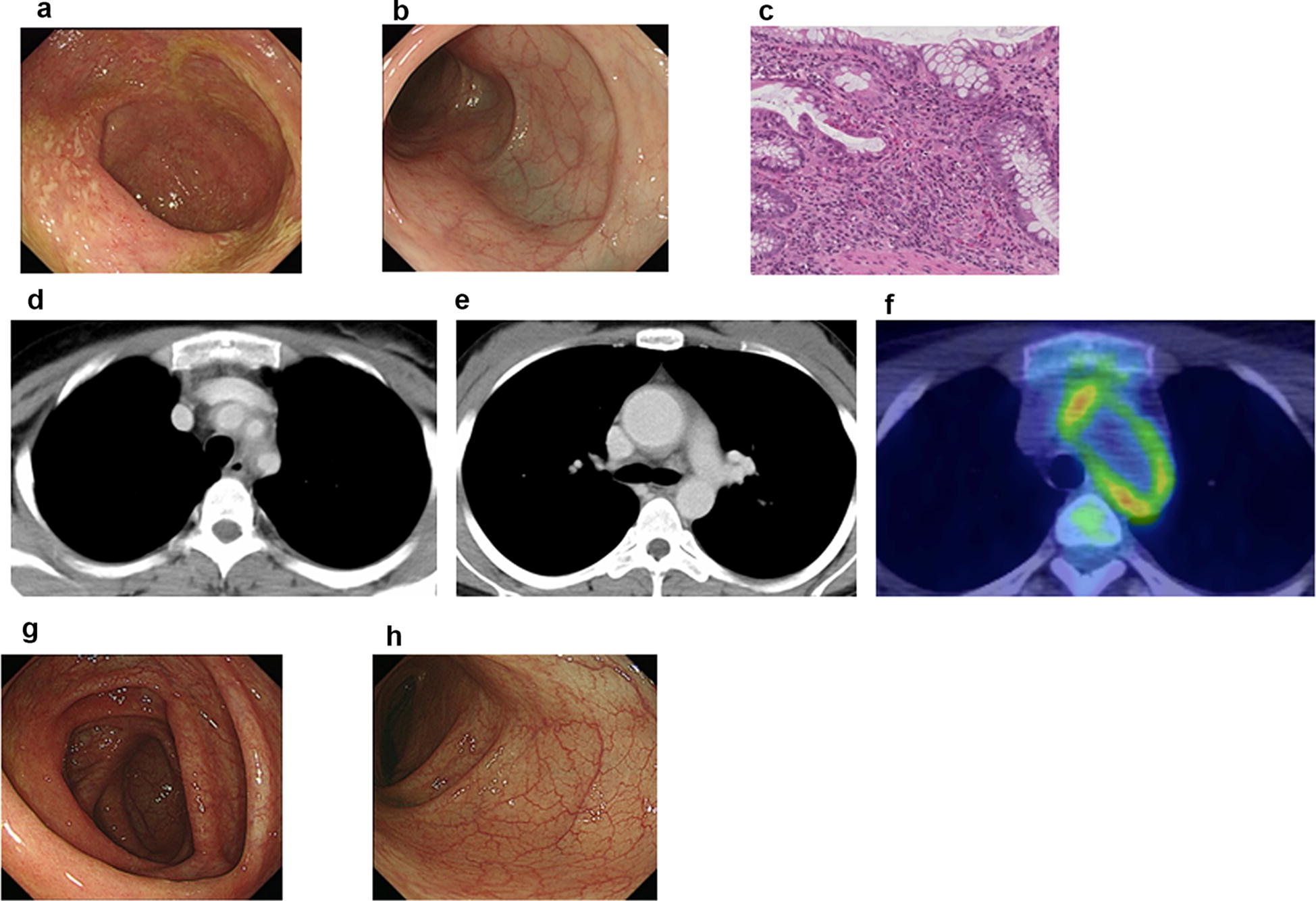
Endoscopic findings of the colon (**a** cecum, **b** sigmoid colon) and pathological findings of the sigmoid colon (**c**) when the case was diagnosed as EGE. Computed tomography scan showing wall thickening of the aortic arch, brachiocephalic artery, right and left subclavian artery and left common carotid artery as well as dilation of the ascending aorta (**d**, **e**). Positron emission tomography (PET) scans showing the FDG uptake along the aortic arch, brachiocephalic artery, right and left subclavian artery and left common carotid artery (**f**). Endoscopic findings of the colon (**g** cecum, **h** sigmoid colon) when the case was diagnosed to have TA

An 18-year-old female had abdominal symptoms of pain and diarrhea. She was diagnosed with unclassified colitis and treated with drugs for controlling the intestinal function, and her abdominal symptoms improved. However, these symptoms recurred 1 year after the first episode. Total colonoscopy was done, which showed edema with multiple erosions in the cecum, ascending, transverse, descending and sigmoid colon. On a pathological examination of biopsied specimens, many eosinophils were found to have infiltrated the colon mucosa (Fig. 1a–c). She was diagnosed with EGE, and the administration of 20 mg/day of prednisolone (PSL) was started. Her symptoms were improved, and the dose of PSL was withdrawn slowly and stopped 3 months after the beginning of PSL treatment. Six months after the end of PSL treatment, she developed a fever and chest discomfort without abdominal symptoms. Laboratory tests showed a white blood cell count of 11,350/µL (neutrophils 72.6%, lymphocytes 21.5%, monocytes 4.7%, eosinophils 0.9%, basophils 0.3%); hemoglobin, 8.7 g/dL; platelet count, 634,000/µL; C-reactive protein (CRP), 13.13 mg/dL; erythrocyte sedimentation rate (ESR), 76 mm/h; serum albumin, 2.3 g/dL; blood urea nitrogen, 12.9 mg/dL; serum creatinine, 0.36 mg/dL; D-dimer 1.40 µg/dL; anti-nuclear antibody, < 40; PR3 ANCA, < 0.1 IU/mL and MPO ANCA, < 0.1 IU/mL. An HLA typing analysis revealed positivity for A2, A31, B35, B39, DR4, DR15, C9 and C7. A 12-lead electrocardiogram revealed no abnormal findings. An echocardiogram revealed dilation of the aorta and moderate aortic regurgitation. Three-dimensional computed tomography (CT) revealed wall thickening of the aortic arch, brachiocephalic artery, right and left subclavian artery and left common carotid artery and dilation of the ascending aorta (Fig. 1d, e). Positron emission tomography (PET) also showed the FDG uptake in the above arteries (Fig. 1f). A diagnosis of TA was made based on the CT and PET findings and elevated CRP and ESR levels, which met the criteria proposed by the Japanese circulation society [4]. She started administration of high-dose prednisolone (1 mg/kg, 40 mg), and her symptoms were improved and the CRP and ESR levels decreased. The dose of PSL was gradually decreased and then withdrawn without relapse. During the period of this hospitalization, she underwent total colonoscopy for the assessment of EGE, which showed no edema and erosion with a little vessel augmentation in the ascending and transverse colon (Fig. 1g, h). On a pathological examination of biopsied specimens, only a few eosinophils had infiltrated the colonic mucosa.

**Fig. 2:**
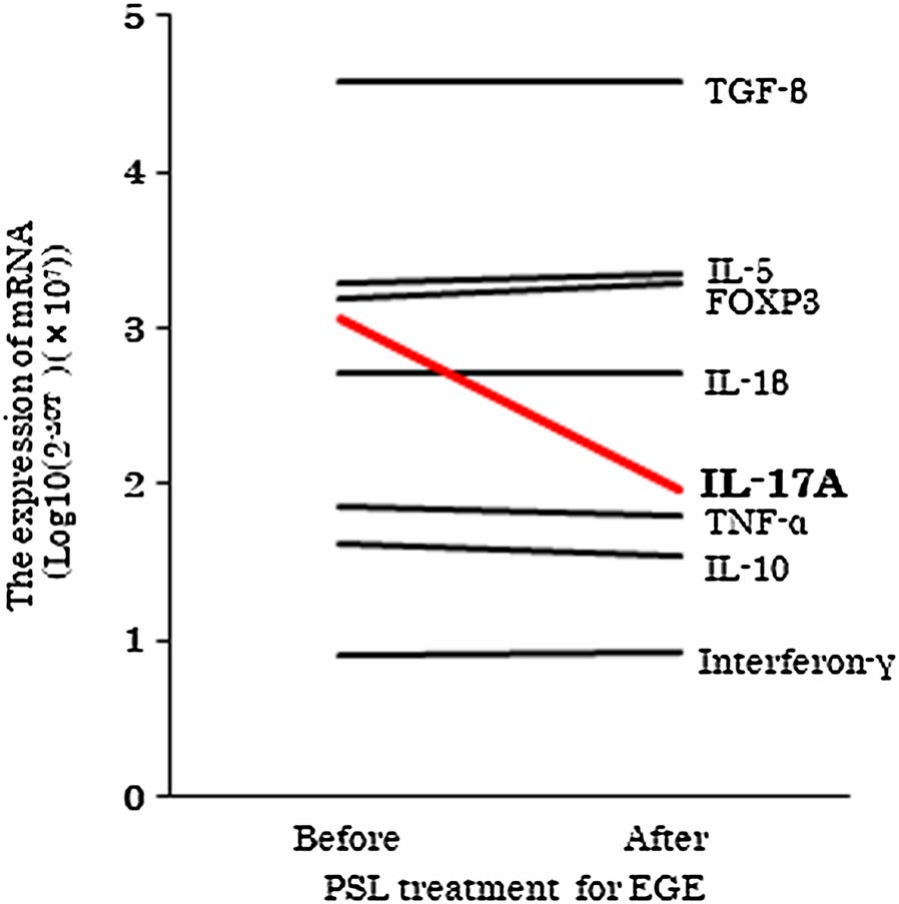
Reverse transcription-polymerase chain reaction (RT-PCR) expression of inflammatory factors associated with Th1 (IL-1β, TNFα and interferon γ), Th2 (IL-4, IL-5 and IL-13), Th17 (IL-17) and regulatory T cells (IL-10, FOXP3) in biopsied specimens of the colon before and after PSL treatment for EGD. IL-4 and IL-13 were not detected

We examined the expression of inflammatory factors associated with Th1 (IL-1β, TNFα and interferon γ), Th2 (IL-4, IL-5 and IL-13), Th17 (IL-17) and regulatory T cells (IL-10, FOXP3) in biopsied specimens of the colon before and after the PSL treatment for EGE using reverse transcription-polymerase chain reaction (RT-PCR) (Fig. 2). RT-PCR showed that the IL-17 mRNA expression was specifically and greatly decreased after the PSL treatment, suggesting that overactivation of Th17 was involved in the pathogenesis of EGE.

## Discussion and conclusions

We herein reported the first case of the coincidence of two rare diseases, TA and EGE. In the present case, TA developed 6 months after PSL treatment for EGE, suggesting two potential mechanisms of TA development. First, TA might have developed after the PSL treatment. Second, TA development might have been masked during PSL treatment for EGE and became apparent only 6 months after the treatment had concluded. Regardless of the mechanism, careful observation is necessary for EGE patients after PSL treatment, and when TA develops, PSL should be administered without delay.

TA is a large-vessel vasculitis characterized by granulomatous necrotizing vasculitis with giant cells. In contrast, EGE is one member within the spectrum of diseases collectively referred to as eosinophilic gastrointestinal disorders. These two diseases are phenotypically different. From immunological perspectives, TH1 and TH17 immunity is thought to be important in driving TA-related inflammation, both systemically and in blood vessels [2, 3]. High levels of TH1 cytokines, such as IL-1β, IL-2, IL-6, IL-12, TNF-α and IFN-γ, and TH17 cytokines such as IL-17A are associated with disease activity. In contrast, the mechanism underlying EGE pathogenesis is thought to be involved in the IgE-mediated and delayed-TH2 type responses in the gastrointestinal mucosa. Preclinical studies have identified a contributory role for cytokines, such as IL-4, IL-5, IL13 and eotaxin chemokines, as well as growth factors, such as TGF-β and EGF [5]. These two diseases are therefore thought to belong to different categories of autoimmune diseases. However, the present case sequentially had two rare diseases, suggesting that some shared factors may have been associated with the pathogenesis of TA and EGE. In addition to TH2 immunity, several immune responses, including TH17 immunity, have been reported to be associated with eosinophilic gastritis [6]. We therefore investigated the mRNA expression of inflammatory factors associated with the activation of Th1, Th2, Th17 and regulatory T cells in biopsied specimens of the colon of the present patient in order to identify the shared mediators involved in the pathogenesis of both TA and EGE. Of note, the mRNA expression of IL-17 greatly decreased in the colon mucosa after PSL treatment, while Th2-related mediators, including IL-5, were not changed during PSL treatment. This suggests that Th17 overactivation, but not Th2, is associated with the pathogenesis of EGE in the present case. Because Th17 overactivation is known to be involved in TA pathogenesis, the overactivation of Th17 may represent a shared mechanism in the pathogenesis of both TA and EGE.

The causes of TA and EGE are still unknown. The present case with TA and EGE suggested that Th17 overactivation may influence the pathogenesis of EGE, which is a new insight concerning EGE pathogenesis. The accumulation of more cases with TA and/or EGE and the analysis of the profiles of inflammation-associated mediators are warranted to clarify the immunological alterations associated with the causes of TA as well as EGE.

## Abbreviations

TA: Takayasu’s arteritis
EGE: eosinophilic gastroenteritis
PSL: prednisolone
CRP: C-reactive protein
ESR: erythrocyte sedimentation rate
CT: computed tomography
PET: Positron emission tomography
RT-PCR: reverse transcription-polymerase chain reaction
